# Phase Delays between Mouse Globus Pallidus Neurons Entrained by Common Oscillatory Drive Arise from Their Intrinsic Properties, Not Their Coupling

**DOI:** 10.1523/ENEURO.0274-24.2024

**Published:** 2024-08-02

**Authors:** Andrea Ortone

**Affiliations:** ^1^The BioRobotics Institute, Scuola Superiore Sant’Anna, Pontedera 56025, Italy; ^2^Department of Excellence in Robotics and AI, Scuola Superiore Sant’Anna, Pisa 56127, Italy

**Keywords:** basal ganglia, cross-correlation function, Fourier transform, perforated patch, phase response curve, synchrony

The external part of the globus pallidus (GPe) is pivotal for the regulation, processing, and control of information within the basal ganglia (BG) network. The GPe is extensively interconnected with the striatum, the subthalamic nucleus, and the internal globus pallidus, forming intricate feedback and feedforward loops. The GPe is primarily composed of two types of GABAergic neural populations, known as arkypallidal and prototypic neurons. Prototypic neurons, the most numerous among these neurons, exhibit high-frequency autonomous spiking activity (30–60 Hz). Under healthy conditions, this activity is largely asynchronous, which is often interpreted as indicative of independent and parallel processing channels within the BG network.

The increase of synchronization in the neural activity of the BG, particularly within the beta frequency range, is significantly associated with the progression of Parkinson's disease (PD; Nini et al., 1995). Over the past two decades, substantial evidence has emphasized the central role of the GPe in generating and propagating pathological oscillations. Key findings include the recording of coherent and uniform local field potentials (LFPs) across the GPe (Goldberg et al., 2004), the impact of optogenetic manipulations of specific nuclei on pathological oscillations, the effects of blocking certain connections (Tachibana et al., 2011), and the intrinsic ability of pallidal neurons to propagate oscillatory drives across a broad frequency range, including the beta band (Wilson and Jones, 2023). Consistent with these observations, the progression of PD leads to an increase in the intensity of pairwise cross-correlation within the beta range between the spike trains of GPe neurons. However, despite the common and coherent input suggested by the homogeneity of LFPs in the GPe, the structure of the cross-intensity function (CIF)— which measures the pairwise cross-correlation between the spike trains of two neurons—exhibits considerable variability in the phase-delays distribution across different pairs of GPe neurons.

To investigate the origins of this variability, E. Olivares, C.J. Wilson, and J.A. Goldberg conducted an experiment where they subjected 16 individual in vitro prototypical GPe neurons to oscillatory currents across a broad frequency range (1–100 Hz; Olivares et al., 2024). For each frequency, they recorded the neurons’ spike trains and calculated the distribution of the phase of the driving current at spike times. When the frequency of the external drive closely matched the neuron's autonomous spiking rate, this distribution became significantly more peaked, indicating that the spikes of each neuron became phase-locked to the external drive. Notably, the locking phase varied significantly among different neurons. To explain this variability, the authors modeled each neuron as a phase oscillator and experimentally derived their individual instantaneous phase response curves (iPRCs). They then theoretically demonstrated that, when entrained by a small coherent external drive, the locking phase of each neuron is fully determined by, and equal to, the angle of the fundamental Fourier component of the neuron's iPRC. This result stems from the orthogonality of the different Fourier components of the iPRC and aligns very well with experimental data.

This finding is crucial as it reveals that the variability in the locking phase of different neurons originates from the intrinsic properties of each neuron and not from population-level effects. However, this method cannot be easily generalized to frequencies of the external drive that deviate significantly from the neuron's autonomous firing rate. In these regimes, the empirical distribution of the phase of the driving current at spike times is more broadly spread across all angles and more closely mirrors the shape of the external drive. As expected, this means that more spikes occur near the maximum of the external drive and fewer spikes close to its minimum, a behavior that can be numerically reproduced by integrating the phase equation with the empirical iPRC.

Given the distribution of the driving phases at spike times for each individual neuron, the authors were able to analytically construct the CIF for each pair of the considered neurons and for any given driving frequency. Qualitatively, we can expect that the phase of the CIF for a given pair of neurons is determined by the difference between the peaks of their respective phase distributions. When this difference is not zero, phase mismatches between the neurons emerge, even in the absence of structural connectivity. Crucially, this conclusion is robust to the introduction of realistic interactions between the neurons. Consistent with previous findings, in silico experiments demonstrated that inhibitory connectivity had strong decorrelating effects on neuronal activity but did not significantly affect the phase distribution of pairwise CIFs. The authors showed that this result holds across different network topologies, including small-world and scale-free networks. However, they did not account for the heterogeneity of neuron populations in the GPe. More structured connectivity patterns, such as those involving arkypallidal neurons, have been shown to potentially alter the dynamical state of the GPe (Gast et al., 2021) and therefore, they may also affect pairwise CIFs.

Together, network topology and the PRCs of individual elements are the primary factors driving the emergence of synchronous oscillatory states in a population of coupled oscillators. The study by Olivares and colleagues suggests that these properties have distinct effects on network dynamics of the GPe nucleus. Specifically, while the amplitude of pairwise CIFs is significantly influenced by inhibitory connectivity and the resulting interactions between GPe neurons, the phase delays of their responses, along with their broad variability, can be entirely explained by the intrinsic properties of the individual neurons. Notably, these phase delays regulate the oscillation frequencies of the resonances that the neuronal population can establish. For this reason, the characterization of the phase-locking distributions and the understanding of their origins represent important steps toward the understanding of the mechanisms underlying the hypersynchronization of the BG network.
